# Association of caries experience and dental plaque with sociodemographic characteristics in elementary school-aged children: a cross-sectional study

**DOI:** 10.1186/s12903-017-0464-4

**Published:** 2018-01-10

**Authors:** Saeed Bashirian, Samaneh Shirahmadi, Shabnam Seyedzadeh-Sabounchi, Ali Reza Soltanian, Akram Karimi-shahanjarini, Farshid Vahdatinia

**Affiliations:** 1 Social Determinants of Health Research Center and Department of Public Health, Hamadan University of Medical Sciences, Hamadan, Iran; 2 Department of Public Health, School of Health, Hamadan University of Medical Sciences, Hamadan, Iran; 3 Dental Public Health, Hamadan University of Medical Sciences, Hamadan, Iran; 4 Department of Biostatistics, School of public health and Modeling of no communicable diseases research center. Hamadan University of Medical Sciences, Hamadan, Iran; 5 Department of Public Health, Hamadan University of Medical Sciences, Hamadan, Iran; 6 Dental research center, Hamadan University of Medical Sciences, Hamadan, Iran

**Keywords:** Caries experience, Elementary school, DMFT/DMFT, Dental plaque

## Abstract

**Background:**

Dental caries among Iranian elementary school children aged 6–12 years continue to rise. To estimate treatment needs and guide health initiatives, current epidemiologic data are required. Such data are currently unavailable for dental health. The purpose of this study was to assess caries experience, dental plaque, and associated factors in elementary school-aged children from Iran.

**Methods:**

In this cross-sectional study, 988 elementary school children aged 7–12 years were selected by multistage cluster sampling. Dental caries was studied using the WHO criteria, dental plaque was examined according to O’Leary index. Data on parental education and occupation, living district, dental pain within the past year, and tooth brushing habits under parental supervision were collected through interviews based on questionnaire. The data were analyzed with descriptive statistics and logistic and linear regression.

**Results:**

The mean (SD) age of the elementary school children was 9.64 (1.73) years. The highest dmft was seen in elementary school children aged 7–8 years 6.53 (4.37) and the highest DMFT and dental plaque was in 12 year olds recorded as 1.17 (1.77) and 51.97 (25.86), respectively. The proportion of decayed teeth in 7 years old elementary school based on dmft index was 80.36%, moreover, the proportion in 12 years old elementary school was 40.17% based on the DMFT index. Age, gender, and dental pain within the past year were significantly associated with DMFT and dmft. The odds of developing dental caries (DMFT) was 1.70 times higher in girls than in boys (*p* < 0.001) and 1.72 times higher in the students that reported dental pain frequently than in those who did not (*p* = 0.005). The chance of developing dental caries (dmft) was 0.47 times lower in girls than boys (*p* < 0.001). Age was significantly correlated with dental plaque such that Plaque Index increased by 2.44 times per one year increase in age (*p* < 0.001).

**Conclusion:**

Results indicated that dental caries experience and plaque formation among elementary school children in Hamadan were high and they were influenced by their sociodemographic factors. The associations found can be used as a helpful guide for planning accurate preventive programs for elementary school children in this region.

## Background

Oral health is a public health issue that may affect both children and adults and impair their quality of life. Despite certain achievements in promotion of oral health in the developed and developing countries, oral diseases are still considered a health issue worldwide [1].

Meanwhile, elementary school children aged 6–12 years old in Iran represent a top priority in Iranian oral health programs because of high prevalence of dental caries and development of permanent teeth at this age group. The importance of maintaining primary teeth and their role in the development and maintenance of permanent teeth as well as the cost-effectiveness of early treatment interventions for permanent teeth in these age groups are other reasons that have prioritized this age group [2]. In this regard Oral Health office in the Ministry of Health and medical science in Iran has started implementing the Program entitled “National oral health promotion program for primary school students” from April 2015 on preventive oral health interventions. The main focus of the oral health development plan is currently on prevention and accurate and up to date research on oral health status of this age group is on their priority list [3].

In Iran, certain factors have been studied that have been correlated with ascending dental caries among elementary school children aged 6–12 years. These factors include inappropriate diet, inadequate fluoride in drinking water, lack of adherence to oral health-related behaviors, and elementary school-age children’s and families’ low levels of knowledge [4–6]. In 2004–2005, each Iranian child aged 5–6 years had 4.3 decayed teeth and in 2011–2012 each school child aged 12 years had 1.62 decayed teeth, increasing to 4.5 and 1.71, respectively, which demonstrates increase in dental caries within less than one decade [2, 7]. According to the report of Iran Ministry of Health and Medical Education in 2011–2012, mean dmft was 5.16 and the mean prevalence rate of dental caries 85.93% among children aged 5–6 years. In addition, the mean DMFT was 2.09 and 3.29 and the mean prevalence rate of dental caries 81.83% and 74.79% among adolescent aged 12 and 15 years, respectively. In addition 84.3%, 75.1%, and 76% of children and adolescent aged 5–6, 12, and 15 years, respectively, were reported to need dental treatments (mainly for dental caries) [2]. In the oral health surveys conducted in Iran, initial lesions have not been counted as needing dental treatment therefore number of dental treatment needs may differ from dental decay reports and this phenomenon is generally seen in epidemiological oral health studies [8].

According to the report of Iran Ministry of Health and Medical Education 2011–2012, in Hamadan mean dmft was 5.64 and the mean prevalence rate of dental caries was 95.12% among children aged 5–6 years which was higher than the country average. In addition, the mean DMFT was 2.05 and 3.46 and the mean prevalence rate of dental caries 88.14%, and 86.89%, among Hamadanian school children aged 12 and 15 years, respectively. While the DMFT index in the 12-year-old elementary school children going to school in Hamadan is roughly the same as the country average, its contribution to caries is higher. The average of this indicator in the 15-year-olds is also higher than the national average [2].

In the pass years few national surveys have been conducted on oral health in Iran which the most recent one was in 2012. Therefore epidemiological information of dental caries and dental plaque in Iran is limited [9] because of different data sources for assessing oral diseases and lack of surveillance system [10]. Although the role of socioeconomic factors on oral health behaviors have been studied and some associations have been found in Iranian elementary school-age children population but we don’t have current and comprehensive information on this area in elementary school-age children [10]. Therefore, the present study was carried out to determine the prevalence of dental caries and plaque index and their association with sociodemographic among elementary school-age children.

## Methods

### Study population and samples

The study population of this cross-sectional study was consisted of the elementary school-age children going to elementary schools in the city of Hamadan located in west of Iran. Data collection was conducted from 20th of March to 20th of May, in 2016. The sample size was estimated for infinite population by using the formula (z^2^_1-α/2_)σ^2^/d^2^ where standard deviation of DMFT index was taken as 2.72 [11]. The required precision of the estimate (d) was set at 22% and Confidence Interval 95%. Using the above-mentioned formula, the sample size was estimated to be 580. After adding the non-response error of 10% and Design effect of 1.5 an additional 300 subjects were included. Thus, 988 subjects were selected for this study.

To select the samples of the study, cluster multistage sampling was performed. To achieve this purpose, first, the list of elementary schools in Hamadan was prepared according to the information provided by the Educational Organization of Hamadan. Then, the elementary schools of the two administrative districts (1 and 2) of the Educational Organization of Hamadan were divided into two groups based on access to health care services, as advantaged (downtown) and non-advantaged (suburbs).

**Fig. 1:**
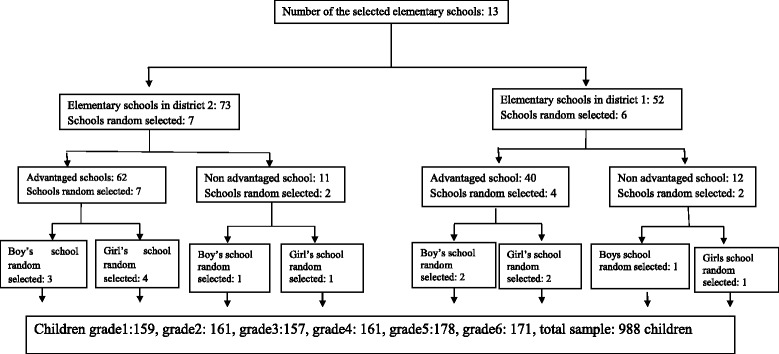
Flow diagram illustration of the sampling process and selection of study subjects from the two general educational districts that 13 schools were finally chosen, and in each school six classes were selected randomly for clinical examination and interview

The number of male and female elementary school-age children enrolled from each administrative district was proportional to the number of schools for boys and girls and the number of the students at each educational grade in that district. Accordingly, seven schools were selected from district 1 and nine schools from district 2 by simple random sampling. Then from the list names of students according to the sample size from all classes in all grades (1st-6th) elementary school children were selected by random sampling. The number of the selected students from each class was proportional to the determined sample size. A total of 988 students were selected. According to sample size calculation required 159, 161, 157, 161, 178, 171 participants respectively in 7, 8, 9, 10, 11, 12 years old; the full sample comprised 988 elementary school-age children (Fig. 1).

The inclusion criteria were age range of 7–12 years old, no previous history of systemic disease, not being under orthodontic treatment. Apart from the day of examination two follow ups were made to schools by the examiner and researcher to complete all the questioners for the selected elementary school-age children in order to prevent coming across missing data. In additional all questionnaires were completed by interview and by the researcher.

Data on demographic factors that would represent individual characteristics were collected through questionnaires by interviewing the elementary school children; it included the sociodemographic characteristics of elementary school children and their families. Dental factors were numerical and were measured through DMFT, dmft and plaque index; Dental pain experience was measured through the question “In the last 12 months, how often have you had a toothache?” (Seldom/Often/Never); Parent Supervision was recorded by asking them to select one of the following options according to their parents’ reactions while they were brushing their teeth: “1. Watch me while brushing my teeth. 2. Do not watch but advise me 3. Never cared. 4. Only my mother watches me.” The replies were then dichotomized into two groups parent supervision = yes (1) and parent supervision = no (0).

The following social and demographic factors were included in the questionnaires: age, sex, parental education categorized nominally as (primary or lower, high school and more than high school), parental occupation was also nominal categories (Worker, government service, self-employment and no job), and the living district was categorized to center and Suburb. Parental occupations, level of education were collected through asking the parents of selected school children.

### Dental examinations

The examinations were conducted by a dental student at the last year of study who was trained and by a faculty member according to World Health Organization guideline [12]. These examinations were conducted according to infection control standards [13]. To conduct each examination, disposable dental mirror, and ball-ended dental explorer, along with a dental plaque disclosing tablet were used. The data were recorded in the WHO standard form [12]. The examinations were conducted in an empty classroom under natural light. All primary and permanent teeth, except for the third molars, of all participants were examined.

Dental plaque was examined according to O’Leary index. The participants were given disclosing tablets to chew and roll over their tongue all over the entire surfaces of their teeth then, medial, distal, lingual, and buccal surfaces were examined by dental mirror and stained areas recorded on the record form [14].

## Data analysis

DMFT and dmft were calculated by counting decayed, filled, and missing permanent and primary teeth [15]. Dental plaque was measured by dividing the number of plaque containing surfaces by the total number of the examined surfaces [16]. The descriptive statistics (mean, standard deviation, frequency, and ratio) of all measured oral health indexes and demographic characteristics were computed.

Previous studies have demonstrated that age, gender, the living district, dental pain experience, parental education and occupation and parent supervision can be predictors of dental caries and dental plaque [17–21]. We wanted to investigate whether the variables included and studied in other studies could predict plaque and DMFT/dmft presence as an outcome variable in elementary school-age children in the city of Hamadan as well. Therefore we included Age, sex, parental education and occupation, living district, dental pain within the past year and tooth brushing under parental supervision; as predicting variables for dental caries and dental plaque (outcome variables) in elementary school children in the city of Hamadan.

Multifactor analysis of ANOVA while controlling for age was used to study the relationship between dt/DT, mt/MT and ft./FT and the sociodemographic variables of the elementary school-age children. Multiple linear regression analysis was employed to analyze the association of various sociodemographic (Age, sex, parental education and occupation, living district, dental pain within the past year and tooth brushing under parental supervision) and outcome variable included plaque index. Multiple logistic regression analysis was executed to test the associations of preset independent/predicting variables (Age, sex, parental education and occupation, living district, dental pain within the past year and tooth brushing under parental supervision) with outcome variables included dental caries (DMFT, dmft), expressed as odds ratios (OR) with 95% confidence intervals (CI). For all analyses, statistical significance was assumed if *P* < 0.05. The regressions were adjusted for age and gender variables. Data were analyzed using the SPSS version 16.

## Results

In total 988 elementary school-age children were examined and interviewed in this study which 503 (50.91%) of them were boys and 485 (49.08%) were girls (Table 1). Mean (SD) DMFT was 0.79 (1.35), mean dmft 3.61 (3.58), and mean dental plaque 46.50 (23.70, Table 1). The boys had 77.5% caries experience in primary teeth and 30% caries experience in permanent teeth and the girls had 68.2% caries experience in primary teeth and 40.8% caries experience in permanent teeth (Table 1). The highest dmft was seen in boys aged 7 years 7.87 (4.36), the highest DMFT in girls aged 12 years 1.43 (2.10), and the highest dental plaque in boys aged 12 years 52.78 (25.71).

**Table 1:** Characteristics of student’s oral health indexes dmft, DMFT and PI according to their demographic variables

Variables	Categories		dmft^a^		DMFT^a^		PI^b^
		N	Mean (SD)	With caries^c^ (%)	Mean (SD)	With caries^c^ (%)	Mean (SD)
Total		988	3.61(3.58)	73.00	0.79(1.53)	35.30	46.51(24.75)
Gender	Boys	503	4.04(3.78)	77.50	0.63(1.17)	30.00	47.08(25.26)
	Girls	485	3.16(3.31)	68.20	0.96(1.49)	40.80	45.92(24.22)
Age(year)	7	153	6.53(4.37)	94.10	0.15(0.54)	9.20	39.00(23.34)
	8	150	5.85(3.30)	93.30	0.70(0.10)	36.70	40.39(20.07)
	9	158	4.91(2.77)	94.30	0.77(1.31)	35.40	47.29 (24.64)
	10	155	3.08(2.73)	80.60	0.82(1.39)	36.10	49.45(24.85)
	11	178	1.69(2.11)	57.90	0.99(1.33)	45.50	48.91(26.02)
	12	194	0.71(1.34)	30.90	1.17(1.77)	48.80	51.97(25.86)
District	Center	571	3.64(3.61)	71.60	0.86(1.44)	36.80	46.70(24.96)
	Suburb	417	3.57(3.55)	74.80	0.69(1.21)	33.30	46.25(24.49)
Dental pain experience	Never	284	2.64(3.42)	58.10	0.79(1.40)	33.10	47.97(26.70)
	Seldom	432	3.32(3.22)	74.80	0.82(1.41)	36.30	46.50(24.35)
	Often	272	5.07(3.84)	85.70	0.74(1.20)	36.00	45.00(23.23)
Father’s Education	≤Primary	58	4.02(3.81)	79.30	0.76(1.21)	34.50	48.87(26.24)
	High School	790	3.65(3.62)	73.20	0.79(1.38)	35.60	46.06(24.89)
	<High School	140	3.22(3.22)	69.30	0.79(1.22)	34.30	48.07(23.35)
Mother’s Education	≤Primary	59	4.54(4.34)	76.30	0.71(1.20)	32.20	50.64(26.06)
	High School	827	3.61(3.57)	73.60	0.80(1.38)	35.40	46.23(24.95)
	<High School	102	3.05(3.04)	65.70	0.77(1.19)	36.30	46.41(22.28)
Father’s occupation	worker	129	3.89(3.81)	76.00	0.78(1.33)	36.40	48.68(25.99)
	Government service	631	3.77(3.64)	75.10	0.78(1.32)	35.30	45.47(24.43)
	Self-employment	223	2.97(3.17)	65.50	0.83(1.44)	34.50	47.32(24.10)
	No job	5	3.80(5.21)	60.00	1.20(1.78)	40.00	85.00(33.54)
Mother’s occupation	worker	8	7.00(3.62)	100.00	0.63(1.40)	25.00	55.50(27.62)
	Government service	101	4.07(3.99)	59.30	0.76(1.35)	40.70	43.19(23.50)
	Self-employment	59	2.93(3.36)	75.20	0.90(1.25)	31.70	49.41(22.08)
	No job	820	3.57(3.53)	73.40	0.79(1.36)	35.50	46.62(25.04)
Parent Supervision	Yes	350	2.47(2.92)	63.40	1.00(1.54)	42.60	49.79(25.63)
	No	638	4.23(3.76)	78.20	0.67(1.22)	31.30	44.71(24.08)

^a^dmft/DMFT is an index to show teeth decayed, missing, or filled tooth

^b^PI denoted that present data for plaque index

^c^dichotomous estimate (proportion of elementary school-age children with any decayed, missing, or filled tooth: dmfT/DMFT >0 in %)

The mean (SD) of decayed teeth was 2.68 (3.05) based on dmft index and 0.33 (0.85) based on the DMFT index (Table 2). The proportion of decayed teeth in 7 years old elementary school children based on dmft index was 80.36%, moreover, the proportion in 12 years old was 40.17% based on the DMFT index.

**Table 2:** Comparison of elementary school-age children dt/DT,mt/MT and ft./FT characteristics according to their demographic variables

Variables	Categories	dmft			DMFT		
		dt^a^ mean (SD)	mt^b^ mean (SD)	ft^c^ mean (SD)	DT^a^ mean (SD)	MT^b^ mean (SD)	FT^c^ mean (SD)
Total		2.68 (3.05)	0.60 (1.12)	0.32 (0.93)	0.33 (0.85)	0.009 (0.13)	0.44 (1.04)
Gender	Boys	2.96(3.27)	0.75 (1.22)	0.31 (0.94)	0.31 (0.85)	0.01 (0.17)	0.29 (0.77)
	Girls	2.38(2.79)	0.45 (0.98)	0.32 (0.92)	0.34 (0.85)	0.004 (0.06)	0.60 (1.23)
*P* value		<0.001	<0.001	0.81	0.54	0.24	<0.001
Age(year)	7	5.24 (4.25)	0.81 (1.22)	0.47 (1.05)	0.04 (0.31)	0.00 (0.00)	0.10 (0.46)
	8	4.21(3.15)	1.00 (1.10)	0.62 (1.22)	0.30 (0.81)	0.00 (0.00)	0.39 (0.75)
	9	3.50(2.36)	0.93 (1.21)	0.46 (1.22)	0.31 (0.79)	0.00 (0.00)	0.44 (1.10)
	10	2.24(2.14)	0.57 (1.30)	0.25 (0.74)	0.36 (0.98)	0.00 (0.00)	0.44 (1.02)
	11	1.20(1.54)	0.30 (0.98)	0.16 (0.69)	0.42 (0.93)	0.01 (0.10)	0.56 (1.06)
	12	0.51(1.00)	0.15 (0.55)	0.03 (0.32)	0.47 (0.98)	0.03 (0.27)	0.65 (1.38)
*P* value		<0.001	<0.001	<0.001	<0.001	0.01	<0.001
District	Center	2.58(3.02)	0.61 (1.17)	0.43 (1.12)	0.34 (0.89)	0.008 (0.15)	0.50 (1.08)
	Suburb	2.81(3.09)	0.58 (1.04)	0.16 (0.55)	0.31 (0.80)	0.009 (0.09)	0.36 (0.96)
*P* value		0.11	0.79	<0.001	0.55	0.88	0.02
Dental pain experience	Never	1.89(2.76)	0.53 (1.20)	0.20 (0.63)	0.31 (0.92)	0.003 (0.05)	0.47 (1.01)
	Seldom	2.44(2.62)	0.54 (1.01)	0.33 (1.03)	0.32 (0.80)	0.01 (0.15)	0.47 (1.15)
	Often	3.87(3.59)	0.78 (1.17)	0.41 (1.01)	0.35 (0.85)	0.01 (0.13)	0.37(0.86)
*P* value		<0.001	0.009	0.01	0.79	0.71	0.58
Father Education	≤Primary	3.32(3.23)	0.51 (1.06)	0.17 (0.50)	0.37 (0.93)	0.00 (0.00)	0.37 (0.87)
	High School	2.72(3.10)	0.64 (1.14)	0.27 (0.89)	0.35 (0.88)	0.01 (0.14)	0.42 (1.03)
	<High School	2.14(2.66)	0.45 (0.99)	0.62 (1.21)	0.17 (0.56)	0.00 (0.00)	0.61 (1.12)
*P* value		0.007	0.16	0.004	0.02	0.62	0.26
Mother Education	≤Primary	3.66(3.71)	0.67 (1.27)	0.20 (0.55)	0.37 (0.92)	0.00 (0.00)	0.33 (0.80)
	High School	2.69(3.05)	0.63 (1.13)	0.29 (0.91)	0.35 (0.88)	0.01 (0.14)	0.43 (1.04)
	<High School	2.03 (2.43)	0.37 (0.88)	0.63 (1.20)	0.15 (0.50)	0.00 (0.00)	0.61 (1.09)
*P* value		0.04	0.15	0.04	0.61	0.93	0.83
Father occupation	worker	2.90 (3.01)	0.72 (1.27)	0.25 (0.86)	0.26 (0.74)	0.00 (0.00)	0.52 (1.04)
	Government service	2.07(2.56)	0.42 (0.92)	0.46 (1.17)	0.22 (0.79)	0.00 (0.00)	0.59 (1.25)
	Self-employment	2.84 (3.13)	0.64 (1.14)	0.28 (0.85)	0.38 (0.89)	0.01 (0.16)	0.37 (0.93)
*P* value		0.01	0.02	0.44	0.12	0.34	0.03
Mother occupation	Government service	2.69(3.02)	0.27 (0.76)	0.55 (1.27)	0.16 (0.53)	0.00 (0.00)	0.72 (1.22)
	Self-employment	2.92(3.42)	0.80 (1.20)	0.34 (1.16)	0.27 (0.72)	0.00 (0.00)	0.48 (1.16)
	No job	2.19(2.75)	0.60 (1.13)	0.30 (0.87)	0.35 (0.88)	0.01 (0.14)	0.42 (1.01)
*P* value		0.93	0.09	0.84	0.62	0.70	0.38
Parent Supervision	Yes	1.82 (2.41)	0.37 (0.84)	0.26 (0.86)	0.42 (1.01)	0.01 (0.19)	0.55 (1.14)
	No	3.14 (3.26)	0.73 (1.22)	0.34 (0.97)	0.28 (0.74)	0.004 (0.06)	0.38 (0.97)
*P* value		<0.001	<0.001	0.10	0.004	0.10	0.07

^a^dt/DT is an index to show teeth decayed

^b^mt/MT is an index to show teeth missing

^c^ft/FT is an index to show filled tooth

The proportions of decayed teeth in female elementary school-age children based on dmft and DMFT indices were 75.31% and 35.7%, respectively, while the proportions in male elementary school-age children were 73.26% and 50% respectively (Table 2). A significant difference was observed between the mean value of decayed primary teeth in the children (1.82 ± 2.41) who were supervised by their parents when brushing their teeth with those (3.14 ± 3.26) who were not supervised by their parents (*P* < 0.001, Table 2). The same results were observed when we assessed the permanent teeth of elementary school-age children. The mean value (SD) for decayed teeth the children who had supervised teeth brushing was 0.42 (1.01), and for those children who had unsupervised tooth brushing was 0.28 (0.74), and the difference between these two was significant (*p* < 0.001, Table 2). The difference between girls (6.60 ± 1.23) and boys (0.29 ± 0.77) in terms of the number of filled permanent teeth was significant (*p* < 0.001, Table 2).

There was a significant association between the number of decayed primary teeth and mother’s education level according to the multifactorial analysis of ANOVA (*p* = 0.005, Table 2). The Tukey post hoc test demonstrated that the significant difference (*p* < 0.05) was between the groups of elementary school-age children whose mothers had an education level lower than primary (3.66 ± 3.71) with those that their mothers had a high school education level (2.69 ± 3.05) and those that their mothers had higher than high school education (2.03 ± 2.43). Whereas, the difference between two groups of mothers, as those with high school education level and those with higher than high school education was not significant (*p* > 0.05).

Age, gender, and dental pain in the past year were significantly associated with DMFT. The chance of developing dental caries (DMFT) was 1.70 times higher in girls than in boys (*p* < 0.001) and 1.72 times higher in the elementary school-age children that had reported dental pain frequently than in those who did not (*p* = 0.005, Table 3).

**Table 3:** Relationship between demographic factors and DMFT/dmft by Multiple Logistic regression analysis

Predictor Variables	DMFT		dmft	
	Adjusted OR^a^ (CI 95%)	*P* value	Adjusted OR^a^ (CI 95%)	*P* value
Age(year)	1.34(1.22–1.47)	<0.001	0.37(0.32–0.44)	<0.001
Gender				
Boy(Reference category)				
Girls	1.70 (1.29–2.24)	<0.001	0.45(0.32–0.65)	<0.001
District				
Center(Reference category)				
Suburb	0.78 (0.59–1.05)	0.11	1.18(0.81–1.70)	0.37
Dental Pain Experience				
Never(Reference category)				
Seldom	1.19(0.86–1.66)	0.28	2.79(1.88–4.14)	<0.001
Often	1.72(1.18–2.53)	0.005	2.19(1.33–3.60)	0.002
Father’s Education				
≤ Primary(Reference category)				
High School	0.87(0.40–1.91)	0.74	0.71(0.27–1.88)	0.49
> High School	0.69(0.27–1.76)	0.44	1.30(0.41–4.09)	0.65
Mother’s Education				
≤ Primary(Reference category)				
High School	1.27(0.58–2.76)	0.54	1.17(0.45–3.01)	0.74
> High School	1.34(0.49–3.62)	0.56	0.87(0.26–2.97)	0.83
Father’s Occupation				
worker(Reference category)				
Self-employment	0.89(0.58–1.36)	0.60	0.90(0.51–1.57)	0.72
Government service	0.80(0.48–1.33)	0.39	0.61(0.31–1.17)	0.14
Mother’s Occupation				
No job(Reference category)				
Self-employment	0.79(0.51–1.25)	0.32	1.02(0.57–1.83)	0.92
Government service	1.18(0.63–2.20)	0.59	0.61(0.28–1.33)	0.21
Parent Supervision				
Yes(Reference category)				
No	0.89(0.66–1.20)	0.47	0.73(0.51–1.06)	0.10

Dependent variable, DMFT, was dichotomized as Carries free = 0, Caries positive = 1

Dependent variable, dmft, was dichotomized as Carries free = 0, Caries positive = 1

CI Confidence interval, OR Odds ratio

^a^The regression was adjusted for sex &age

In addition, age, gender, and dental pain within the past year were significantly associated with dmft. Such that the chance of developing dental caries (dmft) was 0.47 time lower in girls than in boys (*p* < 0.001). Also the probability of developing dental caries was 2.79 and 2.19 times higher in the students who did report frequent and infrequent dental pain compared to those who did not report pain (*p* < 0.05, Table 3). Out of the studied demographic characteristics, only age was significantly correlated with dental plaque (*p* < 0.001, Table 4).

**Table 4:** Relationship between demographic factors and plaque index by multiple linear regressions*

Predictor Variables	B	Std. Error	*P* value
Age	2.44	0.50	<0.001
Gender	−1.31	1.56	0.40
District	−0.91	1.67	0.58
Never experienced dental pain^a^	−1.24	1.87	0.50
Often experienced dental pain^b^	0.25	2.15	0.90
Father’s education			
≤ High school^c^	0.81	4.51	0.85
> High School^d^	3.14	5.41	0.56
Mother’s education			
≤ High school^e^	−4.88	4.43	0.27
> High school^f^	−7.79	5.68	0.17
Father’s Occupation			
Self-employment^g^	−4.16	2.42	0.08
Government service^h^	−3.69	2.90	0.20
Mother’s Occupation			
Self-employment	−1.84	2.52	0.46
Government service^j^	2.77	3.61	0.44
Parent Supervision	−1.83	1.77	0.30

^a^Dental pain experience is a dummy variable taking the value 1 if the respondent is never and zero otherwise

^b^Dental pain experience is a dummy variable taking the value 1 if the respondent is very and little and zero otherwise

^c^Father education is a dummy variable taking the value 1 if the respondent is ≤high school and zero otherwise

^d^Father education is a dummy variable taking the value 1 if the respondent is >high School and zero otherwise

^e^Mother education is a dummy variable taking the value 1 if the respondent is ≤high school and zero otherwise

^f^Mother education is a dummy variable taking the value 1 if the respondent is >high School and zero otherwise

^g^Father occupation is a dummy variable taking the value 1 if the respondent is self-employment and zero otherwise

^h^Father occupation is a dummy variable taking the value 1 if the respondent is government service and zero otherwise

^i^Mother occupation is a dummy variable taking the value 1 if the respondent is self-employment and zero otherwise

^j^Mother occupation is a dummy variable taking the value 1 if the respondent is government service and zero otherwise

*The regression was adjusted for sex &age

## Discussion

This study showed that our population’s current status is not in accordance with WHO’s goal for 2010 of DMFT in 12-year-olds which should be below one [22].

In a national oral health survey conducted on Iranian population in 2011–2012 it was demonstrated that the mean dmft of elementary school children aged 7–8 years was 5.16 nationally and 5.64 in Hamadan [2]. Also the mean DMFT of elementary school children aged 12 years was measured as 2.02 nationally and in Hamadan was 1.93. According to current study, dmft has increased by 1.15 times in elementary school children aged 7 years and DMFT did not change in elementary school children aged 12 years in Hamadan compared to the corresponding figures in national survey in 2011–2012. However 86%, 77%, and 59% of children aged 6 years in Denmark, Sweden, and Wales, respectively, were reported to have no dental caries [23–25]. Additionally the prevalence of dental caries in elementary school children aged 7 years in this study is higher than the mean national caries prevalence and also lower in 12 year olds aged group compared to national figures. Besides that, the prevalence rate of dental caries among elementary school-age children is markedly higher in Iran compared to other countries [2]. High caries experience might be explained by the lack of national preventive programs, insufficient number of pediatric dentists in Iran, and the lack of preventive and educational measures [26, 27]. Also Studies have shown that adequate fluoride concentration in drinking water reduces the prevalence of dental caries (% with dmft /DMFT >0) by 15% and in absolute terms by 2.2 dmft/DMFT [28]. While in Iran various studies have shown that fluoride concentration in Iranian population drinking water is lower than the standard level however there are no community water fluoridation interventions going on in Iran [5].

The results of the present study demonstrated that decayed primary and permanent teeth constituted a large and considerable proportion of DMFT and dmft indices, so that the proportion of filled teeth in dmft index of 7-year old was as low as 6.4% and in DMFT index of 12-year was 55.55%. A high proportion of decayed teeth observed in the present study is similar to those reported by other studies conducted in other developing and undeveloped countries such as India, Afghanistan, and Ethiopia [20, 29, 30] and is higher than those reported in developed countries such as Israel and Spain [31, 32].

Previous studies have demonstrated that the experience of dental caries is a function of dental caries prevalence and the use of dental services such as oral hygiene training, regular dental examinations, dental treatments, and free prevention services [33]. Although such services are readily available for public in developed countries such as Finland, Portugal, and Czech Republic, but they are rarely provided in developing countries [33].

In developing countries like Iran, dental services are commonly expensive, there is no proper insurance for dental services, and in some points of the country do not have any access to such services [26, 27]. Accordingly, the prevalence of dental caries among groups of people with a substandard economic situation is high [33]. The results of the present study are in accordance with previous studies [17, 34] by reaffirming the aforementioned statements so that families with higher education levels had a higher number of filled primary teeth.

The results also demonstrated that the number of filled permanent teeth was higher among elementary school-age children whose fathers had a governmental occupation than others. Essentially, it is expected that self-employed fathers have a higher income, consequently, enabling them to use restorative services more often [35, 36]. Nonetheless, the specific economic situation and the economic downturn in Iran have resulted in a long-term decline in the income of most of the self-employed occupations, and governmental occupations have more security during these years [37].

Similar other studies [17, 38, 39] have demonstrated that elementary school-age children living in the suburb had a lower number of filled primary and permanent teeth compared to those of elementary school-age children living in city center. The reason is somewhat obvious, the elementary school-age children living in the suburb have difficulties in access to dental services and normally have low socioeconomic status [17, 39, 40]. In this regard, we found that 2.1% of parents of elementary school-age children living in the city had an education level lower than primary, whereas, 11% of parents of elementary school-age children living in the suburb had such an education level. Moreover, 3 % of urban elementary school-age children had parents with higher than high school educations while this value for suburb elementary school-age children was equal to 20%. Furthermore, the number of urban elementary school-age children’s fathers with a governmental occupation was 27%, whereas, 15% of suburb elementary school-age children’s fathers had a governmental occupation. Moreover, the prevalence of worker fathers was higher in suburb families than in urban families (18.7% vs, 8.9%).

The present study demonstrated that dental caries was more prevalent in younger age groups, which is consistent with previous studies in Afghanistan, India, Korea, Iran, and Spain [29, 30, 32, 41, 42]. The major contributors to higher caries in primary teeth can be listed as lack of knowledge on efficient preventive behaviors in younger age, inappropriate eating habits such as frequent consumption of sugary foods and snacks, and higher caries resistance in permanent teeth compared to primary teeth [43].

The status of dental plaque in the students (46.5%) in this study was partly consistent with that in developing countries such as Iran and India [42, 44] and was lower than the corresponding index in comparably more developed countries such as Korea and New Zealand [45, 46]. Although tooth brushing is a simple yet effective method to control dental plaque, few students are able to remove their dental plaque via tooth brushing [47]. In this regard oral health education has been reported to help reduce dental plaque in different communities and age groups with different oral health conditions [48].

Meanwhile, schools may be the best place to deliver training on oral health to children, as they provide an appropriate setting for children’s health promotion by offering an educational environment for improving health, through increasing self-esteem, health literacy, self efficacy and sense of control over their lives [49]. The positive messages and practical interventions can be reinforced throughout the consequent years which children are studying in the school. Some believe schools are more influential than families because of positive exposure to teacher support and peer networks [50]. Consistently, several studies have reported promising results on oral health-related interventions in schools [51, 52]. However, in Iran, some schools might receive oral health education infrequently but the majorities do not get codified and continuous training on oral health, and therefore textbooks, workshops and it is suggested that vacant hours in schools could be enriched with oral health training [53].

Consistent with other studies [30, 54, 55], the present study demonstrated that age, gender, and dental pain in the past year were derived predictors of dental caries. However dental plaque was only associated with age after adjusting for other variables’ effects. In other words, the chance of developing dental plaque increased by getting older such that one year increase in age predicted 1.34 times increase in DMFT and 0.37 decrease in dmft. This can be due to several reasons, such as the commutative effect of plaque and calculus by aging, which accelerates the decay and erosion of teeth [56, 57]. The mean value of DMFT was higher in girls than boys. However, the number of decayed teeth was higher in girls than boys. The increase in the number of filled permanent teeth was significant, which can be due to several reasons; permanent teeth begin to grow earlier in girls than boys [58] as well as, females tend to visit dentists and utilize dental services more than males [59]. In addition to the aforementioned reasons, cultural issues should not be overlooked as parents, in Iran, commonly pay more attention to the appearance of their girls than boys [60].

In the present study, it was found that elementary school-age children who experienced toothache more often in the previous year had more decayed primary teeth, filled teeth, and extracted teeth. As recommended by The American Dental Association (ADA), taking care of primary teeth should be started as early as the first primary tooth eruption begins [61]. However, young children do not acquire the required cognitive and functional skills for taking care of their teeth. Consequently, it is the duty of their parents to take care of their kids’ teeth [62].

Unfortunately, Iranian parents do not pay enough attention to the health of primary teeth and delay the dental treatment until the pain becomes intolerable for elementary school-age children. In such situations, tooth extraction is the only option [63, 64]. Similar to previous studies [19, 65], we observed that the number of decayed or extracted primary teeth in elementary school-age children whose parents supervised their tooth brushing was lower than others. However, the number of decayed permanent teeth in these elementary school-age children was higher than others. The high dependency of DMFT on age may be a reason in this regard. Furthermore, as the permanent teeth are completed in the early adolescence and people are strongly opinionated in this period of their lives, they have a high tendency to oppose their parents on their oral hygiene instructions which can lead to a higher prevalence of dental caries [66].

Current evidence demonstrates that parental socioeconomic status is effective on elementary school-age children’s practicing healthy behaviors [54, 67]. Also parental socioeconomic status and elementary school-age children’s oral health have already been reported to be associated [21, 68, 69]; however, the current study did not show any association between parental socioeconomic characteristics and the students’ oral health.

The lack of awareness about the oral hygiene in all layers of society with different socioeconomic status can be an explanation for this finding. The results of our study demonstrated that the proportions of decayed primary teeth in elementary school-age children with higher than high school educated father and mother were 66.45% and 66.55%, respectively. Moreover, there was no significant relationship between mother’s education level and the number of decayed permanent teeth. The lack of awareness and necessary skills regarding taking care of primary teeth can be an influential factor. Similar to previous studies [34, 70] the present study demonstrated that the number of decayed primary teeth had invert association with the mother’s education level. Moreover, the proportion of decayed primary teeth in elementary school-age children whose parents did not supervise them tooth brushing was 74.23%. Parents’ incorrect insistence on treatment rather than prevention may also be another reason. In this regard, the comparison of our results with the results of a national study [2] conducted five years ago demonstrated that the proportion of filled permanent teeth has increased, while the proportion of decayed teeth has remained steady.

On the other hand, elementary school-age children whose parents had a better socioeconomic level (higher than high school educations and governmental occupations) and supervised their elementary school-age children’s oral hygiene had a higher proportion of filled teeth and a lower proportion of decayed teeth. This issue results in a higher prevalence caries experience (dmft and DMFT) in this group of elementary school-age children. Accordingly, it can be postulated that similarity of dental caries experience in elementary school-age children with different socioeconomic levels resulted in a non-significant relationship between oral health status of elementary school-age children and their families’ socioeconomic level [33].

Studies have demonstrated that the socioeconomic level of families can have indirect and mediating effects on the elementary school-age children ‘s oral health status [33, 70], and this could be a reason for observing non-significant relationships between demographic variables and current oral and dental status of elementary school-age children. So the socioeconomic level of a family can affect elementary school-age children’s oral hygiene status indirectly through such factors such as oral health literacy, dietary habits, willingness for brushing teeth, and the use of dental care services [33, 71] which were not not studied in this research project regarding time and resource constraints.

To the best of our knowledge, the current study is the first to provide accurate information about dental caries, dental plaque, and associated factors among students aged 7–12 years in Hamadan, Iran. However, this should be considered in the context of the methodological strengths and limitations of the study. The current study benefits from the following strengths: first, the sample included over 900 locally representative7–12 year-old elementary school children and there was an excellent participation rate; second, distribution of elementary school-age children was well-performed (two different district from the richest to the poorest areas); third, all the oral examinations were performed by one examiner.

The cross-sectional design of this study is one of its limitations because of failing to investigate causal relationship between development of dental caries and low levels of oral health, and underlying factors. Furthermore disclosing tablets stain very thin layers of plaque and might influence amount of plaque scores. However results of this study on plaque numbers were in accordance with the national survey in Hamadan [2] and a study on laboratory modeling of disclosed plaque showed area-based plaque indices can be scored or recorded precisely and with least variability within or between examiners [72]. In addition the outcome of using disclosing tablets has shown to be effective in improving hygiene of patients and has been recommended since the early twentieth century [73].

The use of DMFT index is another limitation of the study. The estimates provided by this index regarding dental caries may be lower than the actual value [74]. The value of this index contains no information about the decay situation, its stage, penetration depth, restoration type, and its situation. Moreover, this index is unable to guide the practitioners in determining the type of required health surveillance, treating decayed teeth, or their periodic examination [8, 75]. Consequently, researchers have recommended utilizing other indices such as international caries detection and assessment system (ICDAS). Because, in Iran, DMFT is still the routine approach for assessing dental caries, and the use of ICDAS needs special training, accurate calibration and specific conditions during clinical examniation in the present study we used DMFT index. Moreover, the DMFT/dmft index is still regarded as a valid approach for assessing dental caries and is the main index used for collecting epidemiological data in many countries [76]. Besides, the World Health Organization (WHO) recommends to use DMFT/dmft index for assessing the prevalence of dental caries in various populations to support the possibility of international comparisons [12].

Receiving socially acceptable responses regarding variables such as parent’s occupation and education level and also positive supervision on elementary school-age children’s tooth brushing was another possible reason that could influence the statistical results and caries experience in elementary school-age children could not be predicted by socioeconomic characteristics of their parents [77, 78]. Although we had tried to control this limitation by checking the schools record books which are kept for each student and their parents’ occupation in all primary schools.

## Conclusion

This study provided accurate epidemiological information on oral health status of 7 to 12 year olds in order to further assist caries prevention and maintenance of oral health interventions. The percentage of elementary school-age children with dental plaque and one or more caries lesions was too high considering the currently ongoing national oral health preventive campaign. The recorded DMFT and PI indexes, in comparison with other countries, illustrate poor oral health, low oral hygiene, and consequently ineffective oral health preventive actions in Hamadan, as well as a need to invest in modern preventive and therapeutic methods.

## Abbreviations

CI: Confidence interval
DMFT: The sum of decayed and filled and missing teeth due to dental caries for permanent teeth
Dmft: The sum of decayed and filled and missing teeth due to dental caries for primary teeth
PI: Plaque index
